# The mediating role of human papillomavirus concern in the relationship between human papillomavirus knowledge and preventive behaviors against sexually transmitted diseases among Turkish women with higher education**

**DOI:** 10.1590/1806-9282.20260700

**Published:** 2026-08-10

**Authors:** Yasemin Yücel, Emine Kiliç Doğan, Özgür Alparslan, Zehra Karataş, İhsan Barış Müldür

**Affiliations:** 1 Sinop University, Faculty of Health Sciences, Department of Midwifery – Sinop, Turkiye.; 2 Tokat Gaziosmanpaşa University, Faculty of Health Sciences, Department of Midwifery – Tokat, Turkiye.; 3 Ministry of Health, Ankara Bilkent City Hospital, Department of Urology – Ankara, Turkiye.

**Keywords:** Human papilloma virus, Health behavior, Preventive health services

## Abstract

**OBJECTIVE:**

The aim of this study was to examine the mediating role of health-related concern in the relationship between human papillomavirus knowledge and behaviors for protection from sexually transmitted diseases among women of higher education.

**METHODS:**

This cross-sectional study included 623 women. Data were collected online using the Personal Information Form, Human Papillomavirus Knowledge Scale, Behaviors for Protection from Sexually Transmitted Diseases Scale, and human papillomavirus Awareness and Concern Level Scale. Data were analyzed using descriptive statistics, Pearson correlation analysis, multiple linear regression analysis, and Structural Equation Modeling with Bootstrap analysis.

**RESULTS:**

The mean scores of the participants were 17.06±6.66 for Human Papillomavirus Knowledge Scale and 79.41±9.38 for Behaviors for Protection from Sexually Transmitted Diseases Scale. Correlation analyses showed that human papillomavirus knowledge was positively associated with awareness (r=0.239; p<0.001), health-related concern (r=0.121; p=0.002), and sexually transmitted disease prevention behaviors (r=0.423; p<0.001). Stigma-related concern was negatively associated with sexually transmitted disease prevention behaviors (r=-0.193; p<0.001). Structural Equation Modeling analyses demonstrated that human papillomavirus knowledge significantly predicted health-related concern (β=0.121; p=0.002), and health-related concern was significantly associated with sexually transmitted disease prevention behaviors (β=0.089; p=0.015). The indirect effect was statistically significant but limited (standardized indirect effect=0.011; p<0.05). The mediation model explained 1.5% of the variance in health-related concern and 18.6% of the variance in sexually transmitted disease prevention behaviors.

**CONCLUSION:**

Health-related concern demonstrated a statistically significant but limited partial mediating role in the relationship between human papillomavirus knowledge and sexually transmitted disease prevention behaviors. The findings suggest that cognitive and affective dimensions should be evaluated separately when examining human papillomavirus-related perceptions and preventive sexual health behaviors

## INTRODUCTION

Human papillomavirus (HPV), one of the most common sexually transmitted diseases (STDs) worldwide and consisting of approximately 200 viruses, is a serious global health problem for women^1,2^. It is reported that nearly 80% of women may be infected with HPV at some point in their lives. Despite its high prevalence, many cases are asymptomatic, with individuals often unaware of the infection, and spontaneous clearance can occur within about 2 years. However, persistent high-risk HPV infections can lead to cervical cancer, which is reported to cause 350,000 deaths worldwide^3^. Risk factors that increase the transmission of HPV, a specific STD, include unprotected sexual intercourse, having multiple sexual partners, and engaging in sexual activity under the influence of alcohol or drugs^4^. Prevention of STDs generally involves maintaining a single sexual partner, consistent condom use, and avoiding contact with bodily fluids. In addition to these measures, vaccination against HPV, which provides protection against multiple strains of the virus, plays a crucial role in HPV prevention^5^. HPV can cause concerns about physiological health problems in women, as well as leading to stigma due to societal perceptions^6^. Therefore, this study aimed to determine the mediating role of HPV concern in the relationship between HPV knowledge level and the prevention of STDs among women with higher education levels. To achieve this objective, the following research questions were formulated to investigate these dynamics among women with higher education levels:

1. Is there a statistically significant relationship between HPV knowledge level and STD prevention behaviors?
2. Is there a statistically significant relationship between HPV knowledge level and HPV concern?
3. Is there a statistically significant relationship between HPV concern and STD prevention behaviors?
4. Does HPV concern mediate the relationship between HPV knowledge level and STD prevention behaviors?

## METHODS

### Study design and setting

This cross-sectional study was conducted using data collected online between March 25 and June 25, 2025. The study population consisted of women with higher education levels In determining the sample size, a correlation coefficient of r=0.151 for HPV knowledge level was adopted based on previous research^7^. Accordingly, the required sample size was calculated as 559 participants, assuming a 95%CI, 5% margin of error, 0.15 effect size, and 95% statistical power. Considering a potential non-response or incomplete data rate of approximately 11.4% (n=64), the study was completed with a total of 623 participants to ensure robust associational analysis.

### Instruments

Data were collected using the “Personal Information Form,” the “HPV Knowledge Scale (HPV-KS),” the “Sexually Transmitted Disease Prevention Behaviors Scale (STD-BS),” and the “HPV Awareness and Concern Scale (HPV-ACLS).”

Personal Information Form: The researcher-developed form, based on the literature, included 12 questions (11 closed-ended, 1 open-ended) covering women’s sociodemographic characteristics and STD/sexual health information^7^.

HPV-KS: The HPV-KS was originally developed by Waller et al. and later adapted into Turkish with established validity and reliability by Bozkurt and Özdemir, comprising 33 items^8,9^. The total score obtainable from the scale ranges between 0 and 33, and higher total scores indicate a higher level of HPV knowledge^9^. The overall Cronbach’s alpha coefficient for the scale is 0.96. In the present study, the Cronbach’s alpha coefficient was found to be 0.91.

STD-BS: Developed by Kılavuz and Yavuz, consists of 21 items and 2 subdimensions, with scores ranging from 21 to 105; higher scores indicate greater STD prevention behaviors^10^. The original Cronbach’s alpha coefficient was 0.91, while it was 0.80 in this study.

HPV-ACLS: Developed by Esencan et al., includes a total of 19 items across three subdimensions (awareness, health concern, and stigma concern)^11^. The lowest possible score on the scale is 0, and the highest possible score is 76. Higher scores on the scale indicate higher HPV awareness-concern^11^. In the present study, the Cronbach’s alpha for the total scale was determined to be 0.840. “The Cronbach’s alpha coefficients for the subdimensions of the scale were found to be 0.730 for the awareness subdimension, 0.770 for the health concern subdimension, and 0.828 for the stigma concern subdimension.”

### Statistical analysis

Statistical analyses were performed using IBM Statistical Package for the Social Sciences (SPSS Statistics 27.0) and Statistics and Analysis of Moment Structures (AMOS 22.0) software. Descriptive statistics were presented as frequencies, percentages, means, and standard deviations. Prior to the main analyses, assumptions of normality and multicollinearity were assessed using skewness-kurtosis values, tolerance values, and Variance Inflation Factor (VIF) statistics. Pearson correlation analysis was conducted to evaluate the linear relationships between the variables. To examine the associations between HPV knowledge, the subdimensions of the HPV-ACLS, and behaviors for protection from STD-BS, multiple linear regression analyses were conducted.

The construct validity of the measurement scales was verified through Confirmatory Factor Analysis (CFA). The model fit indices demonstrated acceptable to good fit for all scales: HPV-KS (x^2^/df=2.919, Comparative Fit Index [CFI]=0.94, Incremental Fit Index [IFI]=0.94, Tucker–Lewis Index [TLI]=0.92, Root Mean Square Error of Approximation [RMSEA]=0.05), HPV-ACLS (x^2^/df=3.904, CFI=0.91, IFI=0.91, TLI=0.90, RMSEA=0.06), and STD-BS (x^2^/df=4.784, CFI=0.93, IFI=0.91, TLI=0.94, RMSEA=0.05).

Following the validation of the measurement models, Structural Equation Modeling (SEM) with Bootstrap analysis was employed to test the mediating role of health-related concern in the relationship between HPV knowledge and STD prevention behaviors. Direct, indirect, and total effects were evaluated, and both standardized and unstandardized path coefficients were reported separately. Statistical significance was accepted as p<0.05.

## RESULTS

When the demographic characteristics of the women participating in the study were examined, their mean age was found to be 25.13±6.65 years, and 74.0% were single. It was determined that 77.7% of the participants had a university degree or higher. Regarding their knowledge of STDs, 73.7% of the women were informed about STDs, while 58.9% possessed a partially sufficient level of knowledge. Additionally, 73.0% of the women were found to have received information on sexual health (Table 1).

**Table 1:** Distribution of descriptive characteristics of the participants (n=623).

Descriptive characteristics	Mean±SD	Min–max
Age (years)	25.13±6.65	18–51
	**Number (n)**	**Percentage (%)**
Marital status		
Married	162	26.0
Single	461	74.0
Family type		
Nuclear family	541	86.8
Extended family	82	13.2
Education status		
Primary/high school	139	22.3
University and above	484	77.7
Income status		
Income less than expenditure	139	22.3
Income equivalent to expenditure income exceeds	383	61.5
Expenditure	101	16.2
Chronic disease status		
Yes	79	12.7
No	544	87.3
Knowledge of sexually transmitted diseases		
Yes	459	73.7
No	164	26.3
Level of knowledge on sexually transmitted diseases		
Sufficient	192	30.8
Partially sufficient	367	58.9
Insufficient	64	10.3
Status of receiving information on sexual health		
Yes	455	73.0
No	168	27.0
Source of sexual health information		
No information	91	14.6
During the training process	393	63.1
Health professionals	65	10.4
From the internet	74	11.9
Should individuals be educated about sexual health?		
Yes	609	97.8
No	14	2.2
At which level should education be given?		
Primary school	65	10.4
Middle school	214	34.4
High school	301	48.3
University	43	6.9
Total	623	100

Mean=average; SD: standard deviation; Min: minimum; Max: maximum.

The mean scores of the women were determined as 17.06±6.66 [min=1–max=31] for the HPV Knowledge Scale (HPV-KS), fort he STD-BS 79.41±9.38 [min=57–max=105] and for the HPV-ACLS 43.37±6.12 [min=24–max=59]. Furthermore, the mean scores for women in the study on the sub-dimensions of the HPV-ACLS scale were 19.54±3.05 [min=8–max=27] for “Health Concern,” 9.94±3.47 [min=0–max=19] for “Stigma Concern,” and 13.88±2.19 [min=5–max=21] for “Awareness.”

Correlation analyses demonstrated that HPV knowledge was positively correlated with awareness (r=0.239; p<0.001), health-related concern (r=0.121; p=0.002), and STD prevention behaviors (r=0.423; p<0.001). In contrast, stigma-related concern was negatively associated with STD prevention behaviors (r=-0.193; p<0.001) and was not significantly correlated with HPV knowledge (r=-0.011; p=0.776). Awareness was not significantly associated with STD prevention behaviors (r=0.063; p=0.114) (Table 2).

**Table 2:** Correlations between study variables and multiple regression analysis predicting sexually transmitted disease prevention behaviors.

Variables			1	2		3	4	5
1. Awareness			1					
2. Health concern			0.124**	1				
3. Stigma concern			0.070	0.405**		1		
4. HPV knowledge			0.239**	0.121**		-0.011	1	
5. STD prevention behavior			0.063	0.139**		-0.193**	0.423**	1
**Predictors**	**B**	**SE**	**β**	**t**	**p**	**%CI**		**VIF**
HPV knowledge	0.523	0.047	0.405	11.183	<0.001	0.431–0.615		1.076
Awareness	-0.169	0.154	-0.040	1.102	0.271	-0.471–0.133		1.073
Health concern	0.622	0.118	0.203	5.256	<0.001	.390–0.855		1.226
Stigma concern	-0.721	0.103	-0.268	-7.003	<0.001	-0.923–0.519		1.203

Dependent variable: STD prevention behavior

**p<0.01. HPV: human papillomavirus; STD: sexually transmitted disease; SE: standard error; CI: confidence interval; VIF: Variance Inflation Factor.

Multiple linear regression analysis showed that HPV knowledge (β=0.405; p<0.001), health-related concern (β=0.203; p<0.001), and stigma-related concern (β=-0.268; p<0.001) were significant predictors of STD prevention behaviors, whereas awareness was not a significant predictor (β=-0.040; p=0.271). Collinearity diagnostics indicated no multicollinearity problem among the variables (VIF=1.076–1.226) (Table 2).

The mediating role of health-related concern was subsequently tested using SEM with Bootstrap analysis. HPV knowledge initially had a significant direct effect on STD prevention behaviors. In the mediation model, HPV knowledge significantly predicted health-related concern, and health-related concern significantly predicted STD prevention behaviors. The indirect effect was statistically significant, indicating partial mediation. However, the direct effect of HPV knowledge on STD prevention behaviors remained stronger than the indirect pathway.

Awareness and stigma-related concern were not included as mediators in the final SEM model because awareness was not significantly associated with STD prevention behaviors and HPV knowledge did not significantly predict stigma-related concern.

The mediating role of health-related concern in the relationship between HPV knowledge and behaviors for protection from sexually transmitted diseases was evaluated using SEM with Bootstrap analysis. In the direct effect model, HPV knowledge had a significant positive effect on STD prevention behaviors (B=0.546, β=0.423, p<0.001), explaining 17.9% of the variance in STD prevention behaviors (R^2^=0.179) (Table 3).

**Table 3:** Structural equation modeling results and path coefficients for the mediation model.

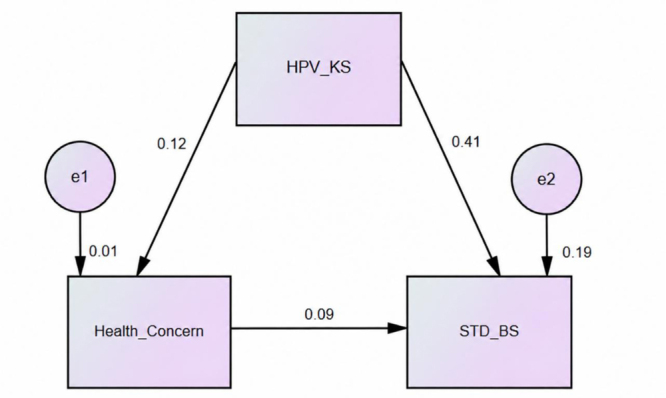							
Paths	B	Standardized β	Standard error	R^2^	Critical ratio	p	Effect type
Direct effect model							
HPV-KS → STD-BS	0.546	0.423	–	0.179	–	<0.001	Direct
Mediation model							
HPV-KS → Health concern	0.051	0.121	0.017	0.015	3.048	0.002	Direct
Health concern → STD-BS	0.272	0.089	0.112		2.433	0.015	Direct
HPV-KS → STD-BS	0.532	0.412	0.047	0.186	11.309	<0.001	Direct
HPV-KS → Health concern → STD-BS	0.014	0.011	–	–	–	<0.05	Indirect
Control variables							
Education level → Health_concern	-0.160	-0.022	0.299	–	-0.535	>0.05	Control
Education level → STD-BS	0.383	0.017	0.831	–	0.461	>0.05	Control

HPV-KS: Human Papillomavirus Knowledge Scale; STD-BS: Behaviors for Protection from Sexually Transmitted Diseases Scale.

In the mediation model, HPV knowledge significantly predicted health-related concern (B=0.051, β=0.121, Composite Reliability [CR]=3.048, p=0.002). In addition, health-related concern had a significant positive effect on STD prevention behaviors (B=0.272, β=0.089, CR=2.433, p=0.015). HPV knowledge continued to have a significant direct effect on STD prevention behaviors after the inclusion of the mediator variable (B=0.532, β=0.412, CR=11.309, p<0.001). The mediation model explained 1.5% of the variance in health-related concern and 18.6% of the variance in STD prevention behaviors (Table 3).

Bootstrap analysis demonstrated that the indirect effect of HPV knowledge on STD prevention behaviors through health-related concern was statistically significant (B=0.014, standardized β=0.011, p<0.05), indicating a limited partial mediating effect. However, the direct effect of HPV knowledge on STD prevention behaviors remained substantially stronger than the indirect pathway (Table 3).

To control for potential confounding effects, educational status was included in the mediation model as a socio-demographic control variable (covariate). The analysis results showed that educational status did not have a statistically significant effect on the mediating variable, Health Concern (B=-0.160, standardized β=-0.022, p>0.05). Similarly, the direct effect of educational status on the ultimate dependent variable, STD-BS was also found to be statistically insignificant (B=0.383, standardized β=0.017, p>0.05). These findings suggest that the underlying pathways and structural mechanisms of the main mediation model are independent of the participants’ educational backgrounds.

## DISCUSSION

HPV is a significant public health problem due to its high prevalence, rapid transmission, and increasing incidence^12^. Therefore, this study investigated the mediating role of HPV concern in the relationship between HPV knowledge and STDs prevention behaviors.

The study revealed that the women included in the research demonstrated an above-average level of HPV knowledge. This finding suggests that the majority of the sample had a high level of education, had received sexual health education, and possessed advanced digital skills, enabling them to access reliable information sources regarding HPV and critically evaluate the credibility of the information. Furthermore, a review of the literature confirms that individuals with higher educational backgrounds and formal sexual health education tend to possess elevated levels of HPV knowledge^13,14^. In addition to these studies supporting our research, it is thought that the level of education and participation in sexual health education may be significant factors influencing the level of HPV knowledge in our study.

The study found that participants had high total STD-BS scores. This result indicates that the women in the sample had very strong preventive health behaviors. Literature shows that individuals who receive sexual health education have significantly higher STD-BS scores^15^. The fact that the majority of women participating in our study had received sexual health education suggests that this can be considered a key determinant of increased STD prevention behavior. Furthermore, this finding indicates that sexual health education can play a critical role in improving women’s preventative health behaviors.

An examination of the scores obtained by the participants from the sub-dimensions of the HPV-ACLS revealed that while health concerns were at a moderate level, stigma concerns were found to be above average. This finding of our research reveals that women may be more concerned about socio-cultural consequences such as exclusion or stigma rather than the health risks that HPV can cause. A review of the literature shows that, especially in cultures where traditional and societal norms prevail, the perception of HPV infection, particularly as an STDs, is more about stigma and criticism from social circles than about health risks^6,16^. Furthermore, it is observed that even with high levels of education, women are not satisfied with this in order to eliminate societal prejudices regarding HPV, and they still experience concern about being stigmatized^6^. Our finding that stigma concerns outweigh health concerns is consistent with the literature. This outcome clearly demonstrates that public health policies and educational interventions regarding HPV should transcend merely providing information on transmission routes and clinical courses; they must critically focus on dismantling socio-cultural taboos associated with the disease and alleviating the psychological and social burden of stigmatization on women.

When the correlation and multiple linear regression analysis results obtained from the study are evaluated together, it was determined that HPV knowledge level is a strong independent variable that positively and significantly predicts protective behaviors against STDs. This finding reveals that an increase in individuals specific knowledge level regarding HPV and, consequently, their accurate assessment of clinical risks (health concern), directly leads them to protective actions and conscious health behaviors. A review of the literature supports this finding^17,18^. Therefore, HPV knowledge level appears to be important for women in terms of protective health behavior. In addition, the significant positive correlations found between HPV knowledge level and awareness, health concerns, and STD prevention behaviors may indicate a cognitive impact on sexual health. This is thought to stem from the fact that increased knowledge among women regarding HPV transmission routes, screening tests, and prevention methods can significantly increase awareness and raise risk perception, leading to health concerns. Furthermore, it is believed that all of these factors contribute to women becoming more conscious about preventive health behaviors^18^.

The study found significant positive correlations between women’s HPV knowledge levels and their awareness, health concerns, and STD prevention behaviors. It is believed that the increased knowledge among women regarding HPV transmission routes, screening methods, and treatment options has influenced their awareness of the issue, and that this awareness has evolved from creating panic-driven fear to a health concern that leads women to adopt preventive approaches. Furthermore, it is thought that all these factors have contributed to increased awareness among women regarding protective behaviors. Indeed, a review of the literature reveals studies that support our findings^17,18^. However, our study shows a negative and significant relationship between women’s fear of stigma and their behaviors in protecting themselves from STDs. This finding suggests that the fact that HPV causes social judgment, exclusion, shame, and labeling for women may lead them to avoid health practices such as using barrier methods, going for gynecological examinations, and undergoing screening, which are presented as steps taken to protect themselves from STDs^19^. The fact that the study did not show a significant relationship between HPV knowledge level and stigma concerns suggests that, due to societal prejudices, women’s concerns about stigma, regardless of their knowledge level, may influence the findings as a social phenomenon. Furthermore, the lack of a statistically significant relationship between HPV awareness and STD prevention behaviors suggests that a difference between women merely hearing the name of HPV and having in-depth knowledge about it creates differences in prevention behaviors.

This study reveals that “health concern” plays a partial mediating role in the effect of HPV knowledge on STDs prevention behaviors. The findings show that HPV knowledge is a key factor triggering protective behaviors in individuals, but this effect is not limited to direct information transfer; it also operates through the individual’s perception of the information as a personal health threat (health concern). The existence of a direct relationship between knowledge and STDs prevention behaviors proves that the basic knowledge individuals acquire about HPV directly translates into preventive actions. However, the inclusion of health-related concern as a mediating variable in the model supports the idea that psychological factors act as “mediators” in the transformation of information into behavioral change. This indicates that information transfer alone is insufficient; behavioral change accelerates when the individual internalizes the information as a threat to their health (health concern). It is noteworthy that even after the inclusion of the health-related concern variable in the model, the direct effect of HPV information on preventive behaviors remains dominant. This finding suggests that the effect of HPV information on behavioral change is not limited to health concern as a mediating variable, but that the information itself is also a strong and independent determinant. Although the sample group in the literature differs from our study, there are studies showing that HPV knowledge increases compliance with preventive measures in individuals^20,21^. Therefore, it can be said that knowledge is important for its transformation into behavioral action. In addition, the HPV knowledge level in our study is a fundamental element in terms of protective behaviors against STDs even without health concerns. In a study conducted on a group with high health knowledge in the literature, the capacity of knowledge to transform into behavior is emphasized^22^. This situation supports the dominance of direct effect in our study, as well as confirming the importance of the competence that knowledge provides to the individual in consciously evaluating the risk and transforming it into behavior. The fact that HPV awareness and stigma concerns were not found to be significant predictors of preventive behaviors in our study indicates that individuals exhibit behavioral change when they develop concrete concerns about their own body health, rather than at a superficial level of awareness. Finally, the lack of a significant effect of education level on the model proves that the mechanism operates through “health concern,” a universal psychological process, rather than socio-demographic differences; this suggests that STD education can yield similar results in broad populations regardless of educational background.

## CONCLUSION

The study found that HPV knowledge levels were above moderate, while behaviors for protection from sexually transmitted diseases were relatively high among the participants. Additional analyses demonstrated that the subdimensions of the HPV Awareness and Concern Level Scale showed distinct relationships with STD prevention behaviors. Awareness was not significantly associated with prevention behaviors, whereas health-related concern demonstrated a positive association and stigma-related concern demonstrated a negative association with STD prevention behaviors.

SEM analyses indicated that health-related concern had a statistically significant but limited partial mediating role in the relationship between HPV knowledge and STD prevention behaviors. However, the direct association between HPV knowledge and prevention behaviors remained substantially stronger than the indirect pathway. These findings suggest that HPV knowledge may be associated with preventive behaviors not only directly but also indirectly through health-related concern. The findings also emphasize the importance of considering cognitive and affective dimensions separately when evaluating HPV-related perceptions and behaviors. Educational interventions provided by healthcare professionals may contribute to improving HPV knowledge and supporting protective sexual health behaviors of women.

## LIMITATIONS AND STRENGTHS

This study has several limitations. First, the sample consisted exclusively of social media users and included a highly educated participant group, which may limit the generalizability of the findings to the broader population of women of reproductive age. In addition, the use of self-reported measures may have introduced social desirability and reporting bias. The cross-sectional design also prevents causal inferences regarding the relationships between HPV knowledge, health-related concern, and STD prevention behaviors. Furthermore, although the mediation model was statistically significant, the indirect effect size was relatively small and should therefore be interpreted cautiously. Despite these limitations, the study has important strengths. To our knowledge, this is one of the first studies to examine the distinct subdimensions of HPV awareness and concern separately within a mediation framework. By differentiating awareness, health-related concern, and stigma-related concern, the study provides a more theoretically nuanced understanding of the relationship between HPV knowledge and STD prevention behaviors.

## Data Availability

The datasets generated and/or analyzed during the current study are available from the corresponding author upon reasonable request.
